# Improved vaccine protection against retrovirus infection after co-administration of adenoviral vectors encoding viral antigens and type I interferon subtypes

**DOI:** 10.1186/1742-4690-8-75

**Published:** 2011-09-26

**Authors:** Wibke Bayer, Ruth Lietz, Teona Ontikatze, Lena Johrden, Matthias Tenbusch, Ghulam Nabi, Simone Schimmer, Peter Groitl, Hans Wolf, Cassandra M Berry, Klaus Überla, Ulf Dittmer, Oliver Wildner

**Affiliations:** 1 Department of Molecular and Medical Virology, Institute of Microbiology and Hygiene, Ruhr-University Bochum, Bldg. MA, Universitaetsstr. 150, D-44801 Bochum, Germany; 2 Institute of Virology, University Hospital Essen, University Duisburg-Essen, Robert-Koch-Bldg., Hufelandstr. 55, D-45122 Essen, Germany; 3 Institute of Cell Biology (Cancer Research), Department of Molecular Cell Biology, University Hospital Essen, University Duisburg-Essen, Hufelandstr. 55, D-45122 Essen, Germany; 4 Institute for Medical Microbiology and Hygiene, University Regensburg, Franz-Josef-Strauss-Allee 11, 93053 Regensburg, Germany; 5 Department of Molecular Virology, Heinrich-Pette-Institute for Experimental Virology and Immunology, University Hamburg,Martinistr. 52, 20251 Hamburg, Germany; 6 School of Veterinary and Biomedical Sciences, Murdoch University, South Street, Perth, 6150, WA, Australia; 7 Paul-Ehrlich-Institut, Division of Medical Biotechnology, Paul-Ehrlich-Str. 51-59, D-63225 Langen, Germany

**Keywords:** Friend virus, interferon alpha subtypes, human adenovirus vectors, human immunodeficiency virus, vaccine

## Abstract

**Background:**

Type I interferons (IFNs) exhibit direct antiviral effects, but also distinct immunomodulatory properties. In this study, we analyzed type I IFN subtypes for their effect on prophylactic adenovirus-based anti-retroviral vaccination of mice against Friend retrovirus (FV) or HIV.

**Results:**

Mice were vaccinated with adenoviral vectors encoding FV Env and Gag proteins alone or in combination with vectors encoding IFNα1, IFNα2, IFNα4, IFNα5, IFNα6, IFNα9 or IFNβ. Only the co-administration of adenoviral vectors encoding IFNα2, IFNα4, IFNα6 and IFNα9 resulted in strongly improved immune protection of vaccinated mice from subsequent FV challenge infection with high control over FV-induced splenomegaly and reduced viral loads. The level of protection correlated with augmented virus-specific CD4^+ ^T cell responses and enhanced antibody titers. Similar results were obtained when mice were vaccinated against HIV with adenoviral vectors encoding HIV Env and Gag-Pol in combination with various type I IFN encoding vectors. Here mainly CD4^+ ^T cell responses were enhanced by IFNα subtypes.

**Conclusions:**

Our results indicate that certain IFNα subtypes have the potential to improve the protective effect of adenovirus-based vaccines against retroviruses. This correlated with augmented virus-specific CD4^+ ^T cell and antibody responses. Thus, co-expression of select type I IFNs may be a valuable tool for the development of anti-retroviral vaccines.

## Background

Type I interferons (IFNs) are major players of the innate immune response, which are produced by virus-infected cells and plasmacytoid dendritic cells. The murine genome comprises 14 type I IFN genes that encode structurally similar proteins of 161-167 amino acids in length. Type I IFN stimulation of a cell results in the expression of hundreds of IFN-regulated genes that mediate an anti-viral state of the cell [1]. In addition, type I IFNs also modulate adaptive immune responses by activating antigen-presenting cells, promoting natural killer cell cytotoxicity and enhancing the proliferation of CD4^+ ^and CD8^+ ^T cells [1]. All type I IFNs bind to and signal through the same receptor IFNAR (IFNα receptor) that consists of the two subunits IFNAR1 and IFNAR2; yet the anti-viral and immunomodulatory effects mediated by individual type I IFN subtypes vary considerably [2,3]. Distinct anti-viral effects of IFN subtypes were demonstrated in several infection models including murine cytomegalovirus, herpes simplex virus, influenza virus and Friend retrovirus infection [4-9].

While the antiviral functions of type I IFNs have been elucidated in detail, and IFN combination therapy is the standard of care in some viral infections like chronic hepatitis B and hepatitis C virus infection [10,11], their potential for modulating adaptive immune responses has only come into focus in recent years. Differing properties of distinct type I IFN subtypes have been described for immunotherapeutic approaches, but have not been systematically characterized for their effects on prophylactic vaccines. In the work presented here, we aimed to analyze type I IFN subtypes for their respective modulating effect on anti-retroviral immunization.

Even after 25 years of intensive research, an effective HIV vaccine remains elusive. Up to now, innumerable vaccine candidates have been developed and evaluated in preclinical models, but only three vaccines have been advanced into efficacy testing in large phase IIB or phase III clinical trials. The vaccination with a protein-based vaccine or adenoviral vectors, aiming exclusively at the induction of antibody responses or cytotoxic T cell responses, respectively, did not result in any protective effect [12,13]. Recently, the vaccination of a community-risk group with a prime-boost combination of protein- and canarypox vector-based vaccines conferred moderate protection and instilled new hope in the field [14]. This data, together with results from animal models [15,16], indicate that for the prevention of HIV infection, both cellular and humoral responses are necessary, and show that it is mandatory to develop means to selectively enhance these responses.

To analyze the protective effect of type I IFN subtypes on adenovirus-based immunization, we employed the Friend virus (FV) model. FV is an immunosuppressive retrovirus complex of the non-pathogenic Friend murine leukemia virus (F-MuLV) and the pathogenic, replication-defective spleen focus forming virus (SFFV). FV infection of susceptible adult mice induces splenomegaly and erythroleukemia and takes a lethal course within a few weeks [17]. The FV infection is regarded as a very useful model for the analysis of immune responses to retroviral infections and for the identification of mechanisms of protection. It was shown that complete immune protection from FV infection requires complex immune responses involving antibodies and CD4^+ ^as well as CD8^+ ^T cells [15]. Previously, we demonstrated that the FV model is very suitable for the development and assessment of novel vectors and strategies for anti-retroviral vaccination. In this model, we showed the benefit of heterologous adenovirus-based prime-boost immunization, which resulted in better protection from FV challenge and enhanced neutralizing antibody responses than the repeated administration of one vector type [18]. Furthermore, we developed a new type of adenovirus-based expression-display vector that not only encodes a transgene, but also presents it on the adenovirus capsid and conferred strong protection from FV challenge infection, correlating with augmented CD4^+ ^T cell and anamnestic neutralizing antibody responses [19].

Using adenoviral vectors encoding F-MuLV Env and Gag proteins co-administered with vectors encoding murine type I IFN subtypes, we aimed to elucidate the effects of particular subtypes on vaccine-mediated protection in the FV model. To verify the results obtained in the FV model, we also performed immunizations of mice with adenoviral vectors expressing HIV Env and Gag-Pol proteins with co-administration of vectors encoding type I IFN subtypes.

## Results

### Enhanced FV immune protection after co-administration of adenoviral vectors expressing FV proteins and specific type I IFN subtypes

We generated E1-, E3-deleted Ad5-based vectors with wild-type or chimeric Ad5/35 fiber encoding murine type I IFN subtypes IFNα1, IFNα2, IFNα4, IFNα5, IFNα6, IFNα9 or IFNβ. The identities of the IFN subtypes were verified by sequencing. and similar expression levels and biological functionality were demonstrated in an established bioassay [20] (data not shown). F-MuLV Env and Gag encoding adenoviral vectors were described previously [18].

**Figure 1:**
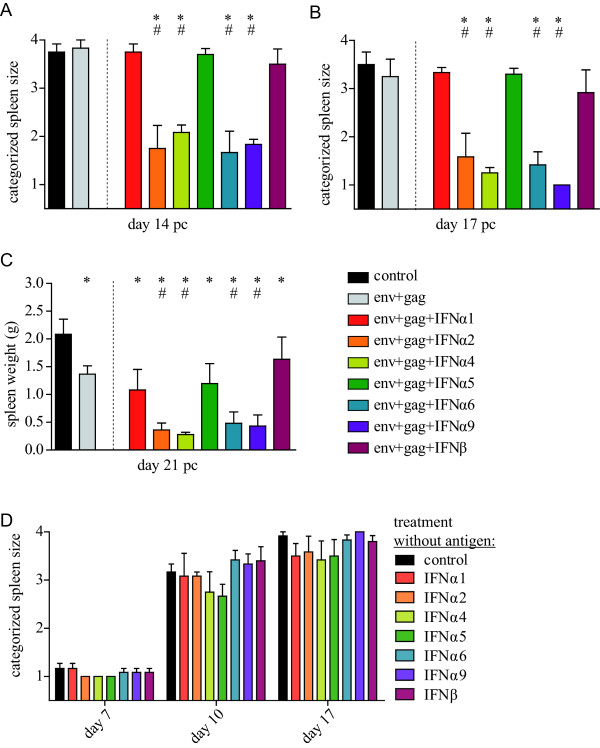
**FV-induced splenomegaly in adenoviral vector immunized mice**. CB6F1 mice were immunized with Ad5 and Ad5F35 based vectors encoding F-MuLV Env and Gag with or without co-administration of a specific vectored type I IFN subtype, as indicated. Ad5-based vectors were used for the prime immunization and Ad5F35 vectors for the boost immunization. Mice of the group "env+gag" received an equal amount of luciferase encoding adenoviral vectors instead of IFN encoding vectors to ensure that the total amount of particles used for immunization was the same in all groups. Three weeks after the boost immunization mice were challenged with FV. Disease progression was monitored by palpation of the spleen twice a week. The categorized spleens of six mice per group on day 14 p.c. (A) and day 17 p.c. (B) are shown (means + standard error of the means). On day 21 p.c. spleens were removed and weighed (C). Statistically significant differences (*P *< 0.05) compared to unvaccinated control mice (*) or mice vaccinated with Env- plus Gag-encoding vectors (#) are indicated. Data are representative of two independent experiments. (D) CB6F1 mice were immunized twice with Ad5 and Ad5F35 based vectors encoding the indicated type I interferons alone and infected with FV three weeks after the second application of IFN vectors. The disease progression was monitored by twice-weekly palpations of the spleen, the graph shows the categorized spleen sizes (mean + standard error of the means) at the indicated time points after FV infection.

Highly FV-susceptible CB6F1 mice were immunized with 1 × 10^9 ^viral particles (VP) each of F-MuLV Env- and Gag-encoding Ad5 vectors and boosted with the same dose of Ad5F35 vectors three weeks later (see Additional file 1, Figure S1 for a schematic outline of the experiment). In contrast to our previous work in which we immunized with 5 × 10^9 ^VP of each vector (1 × 10^10 ^VP total dose) [18,19], this reduced-dose immunization was chosen because it induces only moderate protection on its own, enabling us to analyze the beneficial effect of vectored type I IFN co-administration on vaccine protection. Mice received the adenovirus-vectored F-MuLV antigens co-administered with vectors encoding the selected type I IFN subtypes described above; as a control, one group of mice received the adenoviral vectors encoding F-MuLV Env and Gag and were co-administered vectors encoding luciferase as an irrelevant transgene in order to administer equal amounts of adenoviral particles to all mice. Three weeks after the boost immunization the mice were challenged with FV and the spleen size as a surrogate marker for disease progression was monitored by abdominal palpation. While the immunization of mice with the reduced dose of F-MuLV Env- and Gag-encoding vectors alone did not result in significant protection against initial splenomegaly, co-administration of adenoviral vectors encoding IFNα2, IFNα4, IFNα6 or IFNα9, but not IFNα1, IFNα5 or IFNβ, resulted in significant reduction of FV-induced splenomegaly (*P *< 0.05; shown in Figure 1A and 1B for days 14 and 17 post-challenge (p.c.)). Improved protection after co-administration of the four IFN subtype vectors was confirmed when animals were sacrificed and spleen weights were measured on day 21 p.c. (Figure 1C). At this time point, the spleen weights of all vaccinated mice were significantly lower than of unvaccinated control mice demonstrating a moderate protective effect of the low-dose vaccination with F-MuLV Env- and Gag-encoding Ad5 and fiber-chimeric Ad5F35 vectors. However, protection against splenomegaly was significantly improved when mice had been co-administered vectors encoding IFNα2, IFNα4, IFNα6 or IFNα9 (*P *< 0.05).

To ascertain that the observed effects of type I IFN co-administration were due to a modulation of the immune response to the vaccination and not to a direct antiviral effect of residual IFN expression, mice were administered type I IFN encoding vectors alone and challenged afterwards with FV according to the same scheme. Here, no differences in the control of FV-induced disease were observed (Figure 1D).

### Co-administration of specific type I IFN subtypes mediated improved control over viral replication in vaccinated mice

**Figure 2:**
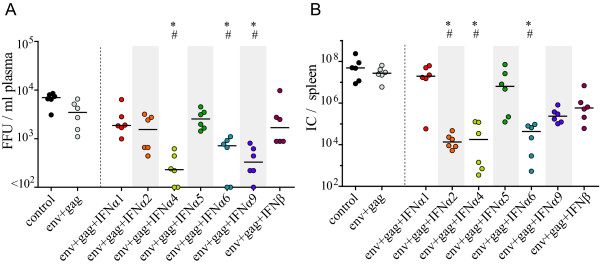
**Viral loads after FV challenge infection of vaccinated mice**. CB6F1 mice were prime- and boost-immunized with Ad5 and Ad5F35 based vectors, respectively, encoding F-MuLV Env and Gag with or without co-administration of a specific vectored type I IFN subtype, as indicated. Three weeks after the boost immunization mice were challenged with FV. Viral loads in the plasma of FV-infected mice were analyzed on day 10 p.c. (A) shows viremia levels as FFU/ml, median values are indicated by lines. On day 21 p.c. the viral loads in spleen were analyzed (B), the graph shows the viral load as IC/spleen, the horizontal lines mark median values. Statistically significant differences (*P *< 0.05) compared to unvaccinated control mice (*) or mice vaccinated with Env- plus Gag-encoding vectors (#) are indicated. Each dot represents an individual mouse. Data are representative of two independent experiments with similar results.

To determine whether co-administration of type I IFNs resulted in improved control over virus replication after FV challenge, viral loads in plasma of animals immunized with F-MuLV Env and Gag encoding vectors with or without co-administration of IFN subtype encoding vectors were analyzed at 10 days after FV infection (Figure 2A). Vaccination with Env- and Gag-encoding vectors alone only slightly reduced acute viral loads but a significant reduction in viral titers was found in animals after co-administration of adenoviral vectors encoding the subtypes IFNα4, IFNα6 or IFNα9 (*P *< 0.05). Some of the mice from these three groups even had viral loads below the detection limit of the assay. Co-administration of vectored IFNα2 also reduced the plasma viremia level of some mice compared to F-MuLV Env and Gag vaccinated mice but this reduction was not statistically significant.

In addition to acute viremia levels, numbers of infectious cells in the spleens of vaccinated mice were determined 21 days p.c. (Figure 2B). Co-administration of adenoviral vectors encoding IFNα2, IFNα4 or IFNα6 resulted in a significant reduction of spleen viral loads compared to both unvaccinated mice or mice vaccinated with Env- and Gag-encoding vectors alone (*P *< 0.05); the reduction in mean spleen viral loads was more than 1000-fold. After co-administration of vectors encoding IFNα9 or IFNβ, the mean viral loads in spleens of mice were also reduced more than 100-fold compared to unvaccinated or Env- and Gag-vaccinated mice, but the differences did not reach statistical significance (*P *> 0.05). No adjuvant effect on vaccine protection against FV was found for IFNα1 and IFNα5.

### IFNα2, 4, 6 and 9 co-expression enhanced vaccine-induced CD4^+ ^T cell responses

**Figure 3:**
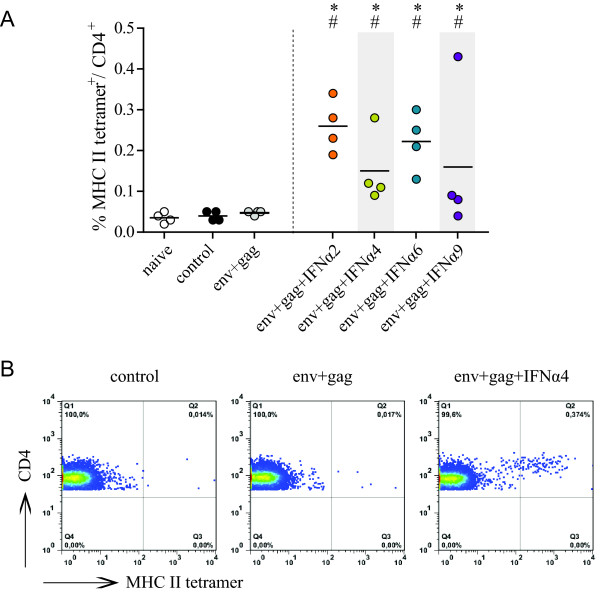
**Vaccine-induced F-MuLV Env-specific CD4**^**+ **^**T cell responses**. CB6F1 mice were prime- and boost-immunized with Ad5 and Ad5F35 based vectors, respectively, encoding F-MuLV Env and Gag with or without co-administration of a specific vectored type I IFN subtype, as indicated. Three weeks after the boost immunization mice were challenged with FV and the F-MuLV Env-specific CD4^+ ^T cell response was analyzed 3 days p.c. by staining with MHC II tetramers presenting an F-MuLV Env gp70-derived epitope. (A) The graph shows the percentage of MHC II tetramer^+ ^CD4^+ ^T cells, the line designates the mean value. Statistically significant differences (*P *< 0.05) compared to unvaccinated control mice (*) or mice vaccinated with Env- plus Gag-encoding vectors (#) are indicated. Each dot represents an individual mouse. Data are representative of two independent experiments with similar results. (B) Representative dot plots from an unvaccinated mouse and mice vaccinated with env+gag or env+gag+IFNα4 are shown.

To elucidate the immunological mechanisms leading to improved protection after co-administration of specific type I IFN subtypes, we analyzed the virus-specific T cell response in mice vaccinated either with Env- and Gag-encoding vectors alone or in combination with vectors encoding the four subtypes that improved protection (IFNα2, IFNα4, IFNα6 or IFNα9). Mice were vaccinated and challenged as described before and class I and II tetramer staining was performed at 3 days p.c. to quantify T cell responses (see Additional file 1, Figure S1 for a schematic outline of the experiment). For FV, only one H2-D^b^-restricted CD8^+ ^T cell epitope, the GagL epitope [21], has been identified so far. However, this epitope is not processed in cells infected with the F-MuLV Gag-encoding adenoviral vector used in this study (data not shown). Therefore, it was not surprising that no FV-specific CD8^+ ^T cells were detected with class I tetramers in any of the vaccinated mice (data not shown). In contrast, shortly after FV challenge F-MuLV Env-specific CD4^+ ^T cells could be quantified using MHC II tetramers presenting an F-MuLV gp70 epitope [22]. No tetramer^+ ^CD4^+ ^T cells were detectable in unvaccinated mice and in mice vaccinated with Env and Gag alone (Figure 3). In contrast, virus-specific CD4^+ ^T cells were found in all mice that were co-administered vectors encoding IFNα2, IFNα4, IFNα6 or IFNα9 (with only one exception in the IFNα9 group; *P *< 0.05; Figure 3A). Representative dot plots are shown in Figure 3B. Thus, these particular IFN subtypes significantly augmented FV-specific CD4^+ ^T cell responses.

### The role of T cells in IFN subtype mediated enhanced vaccine protection

**Figure 4:**
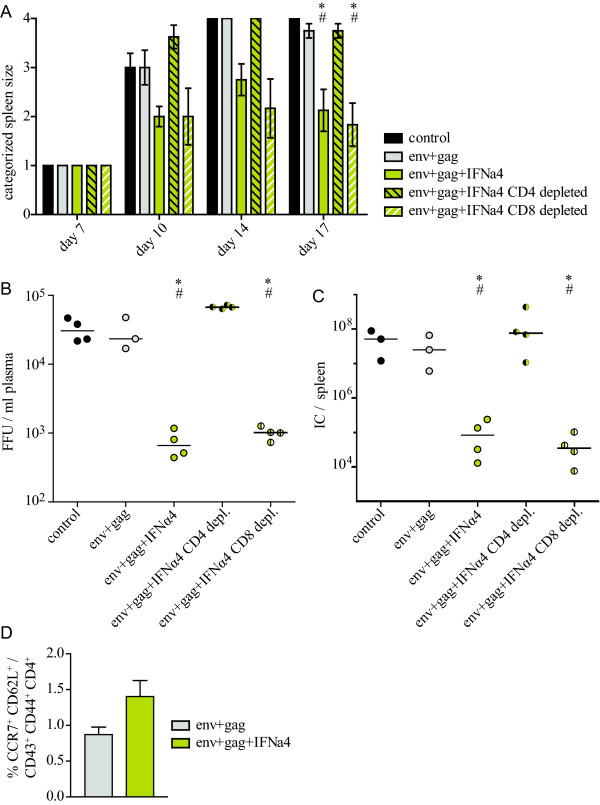
**Depletion of CD4**^**+ **^**and CD8**^**+ **^**T cells during vaccination**. CB6F1 mice were vaccinated with adenoviral vectors encoding F-MuLV Env and Gag with or without co-administration of vectored IFNα4 as described before. On day -3, -1, +1, +3, and +5 of vaccination, mice were injected i.p. with antibodies against CD4 or CD8 to deplete the respective T cell subset. After FV challenge infection, spleens were palpated twice a week to monitor disease progression (A). Viral loads in plasma were determined on day 10 p.c. (B), viral loads in spleen were analyzed on day 21 p.c. (C). For the analysis of T cell induction, mice were immunized once with the indicated vectors and the expression of CD43, CD44, CD62L and CCR7 on CD4+ T cells in the draining lymph nodes was analyzed 10 days after immunization (D). The graphs show data of four mice per group. Statistically significant differences (*P *< 0.05) compared to unvaccinated control mice (*) or mice vaccinated with Env- plus Gag-encoding vectors (#) are indicated.

Previous work indicates that both CD4^+ ^and CD8^+ ^T cell responses contribute to vaccine protection against FV [15]. However, only FV-specific CD4^+ ^T cell responses were detected in the current vaccine study. To elucidate the impact of CD4^+ ^and CD8^+ ^T cells on vaccine-mediated protection after co-administration of IFN subtypes, T cell depletion experiments were performed (see Additional file 1, Figure S1 for a schematic outline of the experiment). IFNα4 co-administration was selected to perform these experiments because it was the subtype that mediated the strongest reduction in viral loads in vaccinated mice (Figure 2). Depletion of CD8^+ ^T cells during the time of vaccination did not result in reduced protection in mice inoculated with vectors encoding Env- and Gag and IFNα4 (Figure 4). This indicates that in the absence of CD8^+ ^T cells reactive to the immunodominant GagL epitope, also no CD8^+ ^T cells of other, unknown specificity played a major role in vaccine-mediated protection. However, depletion of CD4^+ ^T cells had a profound effect on protection. The depletion abolished the protective effect of the Env/Gag/IFNα4 vaccine completely as indicated by severe splenomegaly (Figure 4A) and high plasma (Figure 4B) and spleen (Figure 4C) viral loads in these mice. Thus, the CD4^+ ^T cell response in Env and Gag vaccinated mice, which was augmented by co-administration of IFNα4 (Figure 3) was absolutely critical for vaccine protection.

To analyze the kinetics of the improved CD4^+ ^T cell response, we immunized mice once with Ad5.env+gag or Ad5.env+gag+IFNα4 and analyzed activation of CD4^+ ^T cells in the draining lymph nodes at 3, 7 or 10 days after immunization. While we did not see any difference between mice from the two groups in the percentage of activated CD4+ T cells with effector phenotype (CD43^+ ^CD44^+ ^CD62L^- ^CD4^+ ^T cells; data not shown), at 10 days after immunization the mean percentage of activated CD43^+ ^CD44^+ ^CD4^+ ^T cells with a entral memory phenotype (CCR7^+ ^CD62L^+^) was increased in mice co-administered IFNα4 (Figure 4D), suggesting a role of IFNα4 in CD4^+ ^T cell memory formation.

### Enhanced antibody titers after co-administration of specific type I IFN subtypes

**Figure 5:**
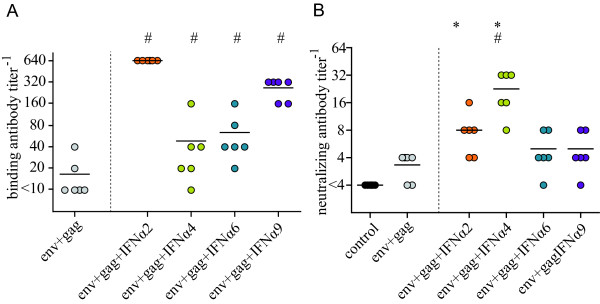
**Vaccine-induced FV-specific antibody response**. CB6F1 mice were prime- and boost-immunized with Ad5 and Ad5F35 based vectors, respectively, encoding F-MuLV Env and Gag with or without co-administration of a specific vectored type I IFN subtype, as indicated. Binding antibodies were analyzed 18 days after boost immunization (A), whereas neutralizing antibodies were analyzed 10 days after FV challenge infection (B). The graphs show the reciprocal titers and horizontal lines mark the mean values. Statistically significant differences (*P *< 0.05) compared to unvaccinated control mice (*) or mice vaccinated with Env- plus Gag-encoding vectors (#) are indicated. Each dot represents an individual mouse. Data are representative of two independent experiments with similar results.

Virus-specific antibodies have been shown to play an important role in vaccine protection against FV infection [15,23]. Therefore, we analyzed the humoral immune responses to vaccination at 18 days after the boost immunization as well as 10 days p.c. After Env- and Gag-vaccination, only two out of six mice had developed detectable F-MuLV-binding antibodies after the boost immunization (Figure 5A), whereas all mice that had received vectors encoding IFNα2, IFNα4, IFNα6 or IFNα9 had significantly higher mean F-MuLV-binding antibody titers (*P *< 0.05), with the highest titers found in mice that had been co-administered vectors encoding IFNα2 and IFNα9. FV neutralizing antibodies were not detected in any of the vaccinated mice (data not shown). However, at ten days after FV challenge, most vaccinated animals showed low titers of F-MuLV-neutralizing antibodies (Figure 5B), which were only increased after co-administration of IFNα2- and IFNα4-encoding vectors compared to the group of Env- and Gag-immunized mice. While this increase was statistically significant for IFNα4 (*P *< 0.05; Figure 5B), it did not reach statistical significance for IFNα2.

### Cellular immune responses to an adenovirus-based HIV vaccine were improved by co-administration of select type I IFN subtypes

To determine whether the adjuvant effect of type I IFN subtypes also applied to vaccination against HIV, we analyzed immune responses to an HIV vaccine in mice using adenoviral vectors expressing HIV Env and Gag-Pol alone or in combination with vectors encoding IFNα2, IFNα4, IFNα6 or IFNα9. For the analysis of cellular immune responses, BALB/c mice were immunized once with Ad5-based vectors and spleens were removed two weeks later (see Additional file 1, Figure S1 for a schematic outline of the experiment) to determine cytokine production by CD4^+ ^and CD8^+ ^T cells after *in vitro *restimulation with HIV Gag derived peptides that have been described before to be relevant T cell epitopes in BALB/c mice [24,25].

**Figure 6:**
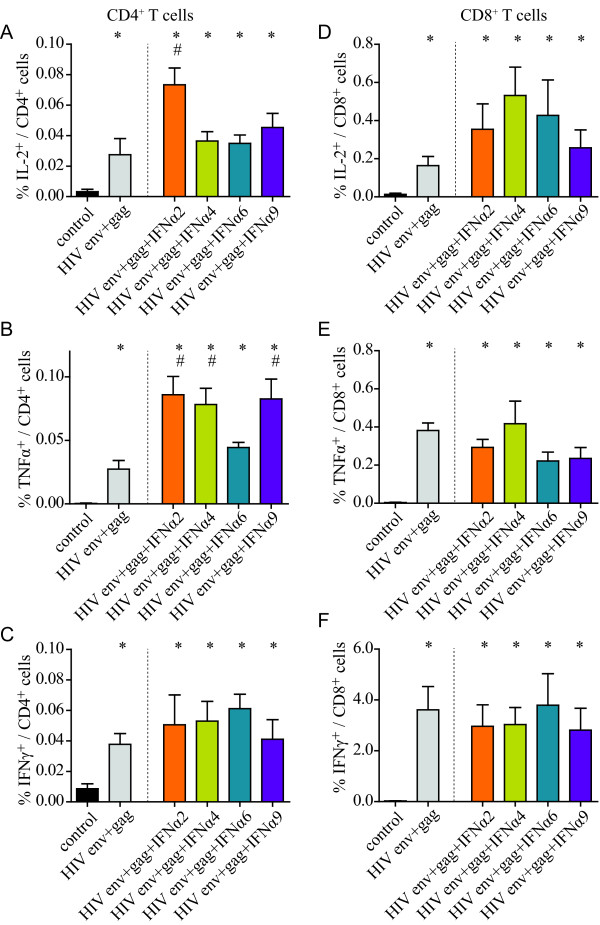
**HIV Env- and Gag-specific T cell responses after adenovirus-based vaccination**. Two weeks after a single immunization with HIV Env and Gag encoding Ad5-based vectors in combination with Ad5 vectors encoding specific type I IFN subtypes, spleens were removed and after *in vitro *restimulation of spleen cells with HIV Gag-derived peptides the expression of IL-2, TNF-α and IFN-γ by CD4^+ ^(A-C) and CD8^+ ^T cells (D-F) was analyzed. The graphs show mean percentages with standard error of the means for six mice per group. Statistically significant differences (*P *< 0.05) compared to unvaccinated control mice (*) or mice vaccinated with Env- plus Gag-encoding vectors (#) are indicated. Data were acquired in two independent experiments.

A significant induction of CD4^+ ^T cells producing IFNγ, TNFα or IL-2 by the HIV antigen encoding adenovirus-based vaccine was observed in all mice (*P *< 0.05; Figure 6 A-C). Compared to Env- and Gag-Pol-immunization alone, significantly higher mean percentages of HIV Gag-specific CD4^+ ^T cells producing IL-2 (only for IFNα2) or TNFα after restimulation were found in mice that had been co-administered vectors encoding IFNα2, IFNα4 or IFNα9 (*P *< 0.05). In contrast, IFNα6 had no effect on HIV Gag-specific CD4^+ ^T cell responses. In addition, the IFNγ expression by CD4^+ ^T cells was not changed by any of the four type I IFN subtypes. Similar results were also obtained when mice were immunized twice with a prime boost immunization protocol using Ad5-based vectors for priming and Ad5F35-based vectors for boosting, resembling the FV experiments. Again IFNα2, IFNα4 or IFNα9 enhanced CD4^+ ^T cells responses but in these experiments the differences between the groups were not as pronounced as after only one vaccination (data not shown).

Similar to the CD4^+ ^T cell response, the HIV Env- and Gag-Pol-immunization induced cytokine producing CD8^+ ^T cells, which responded specifically to restimulation with Gag-derived peptides (Figure 6 D-E). The mean percentages of cytokine producing CD8^+ ^T cells were higher than those for CD4^+ ^T cells. However, after co-delivery of IFNα2, IFNα4 or IFNα6 only slightly enhanced mean percentages of IL-2 producing HIV Gag-specific CD8^+ ^T cells were detected than in Env- and Gag-Pol-immunized mice and the difference did not reach statistical significance. Expression levels of TNFα and IFNγ were comparable in all immunized mice irrespective of IFN co-administration, with very high percentages of IFNγ-producing CD8^+ ^T cells in all groups. Similar results were obtained when CD8^+ ^T cells were restimulated with an Env-derived epitope peptide (data not shown).

These data underline the impact of specific type I IFNs on vaccine-induced CD4^+ ^T cell responses, whereas only little effect on the CD8^+ ^T cell response could be demonstrated.

## Discussion

Type I IFN subtypes have distinct immunomodulatory and antiviral properties, which implies that they have different potentials for immunotherapeutic applications [4-9] and emphasizes the need for careful subtype selection for infectious disease treatment. Type I IFNs have also been used to improve the efficacy of experimental vaccines including protein [26], DNA [8,27-30] and viral vector vaccines [31-33], but a systematic approach to study the adjuvant efficacy of different IFN subtypes has not been undertaken so far. In this study, we analyzed seven adenovirus-vectored type I IFN subtypes for their effect on an adenoviral vector-based anti-retroviral vaccination. As expected, the effect on vaccine efficacy of the IFN subtypes differed greatly. While the co-administration of adenoviral vectors encoding IFNα2, IFNα4, IFNα6 and IFNα9 led to improved control over FV-induced disease and strongly reduced viral loads, no or only slight effects were observed after co-administration of the subtypes IFNα1, IFNα5 and IFNβ. All four IFN subtypes that improved vaccine protection enhanced virus-specific CD4^+ ^T cell and antibody responses.

The importance of the FV-specific CD4^+ ^T cell response for vaccine protection was emphasized by a depletion experiment, in which vaccine-mediated protection was completely abolished when CD4^+ ^T cells were depleted around the time point of vaccination. The kinetic analysis of the early activation of B cells and CD4^+ ^T cells after vaccination suggests that co-administration of IFNα4 might influence the CD4^+ ^memory T cell formation, whereas no differences in overall activation of CD4^+ ^or CD8^+ ^T cells with effector phenotype or B cells was found (data not shown). An improved central memory CD4^+ ^T cell response might be critical for enhanced virus-specific cellular and humoral recall responses after virus challenge, as similar findings have been reported for FV infected mice [34].

The importance of CD4^+ ^T cell responses for vaccine-induced protection from FV infection has been demonstrated before in mice vaccinated with an attenuated F-MuLV and in vaccination studies with peptides containing CD4^+ ^T cell epitopes [35,36]. In the attenuated vaccine experiments it was suggested that the CD4^+ ^T cells mainly provided help for B cells and CD8^+ ^T cells rather than exerting direct effector functions [15,37]. It was also shown in FV infected mice that the CD4^+ ^T cell response is crucial for efficient induction of virus-specific antibodies [34]. These findings correspond well with our data, as we observed a correlation of CD4^+ ^T cell induction and antibody responses in mice vaccinated against FV.

The important role of antibodies for protection from FV infection has also been demonstrated before [15,37]. It is noteworthy that in our challenge experiment, mice that were co-administered IFNα4 had the strongest anamnestic neutralizing antibody response and also the lowest acute viral load in plasma. While this antibody response might be influenced by the augmented CD4^+ ^T cell response, a direct effect of the type I IFNs on antibody induction is conceivable, as it has been shown that type I IFNs enhance primary antibody responses, promote isotype switching and can increase B cell survival [38,39].

In mice immunized with peptides representing CD4^+ ^T cell epitopes, improved maturation of neutralizing antibodies [36], but also direct effector function of vaccine-induced CD4^+ ^T cells have been reported [35]. Also in FV-infected mice, direct anti-viral effects of CD4^+ ^T cells have been documented [34,40,41], which were found to be mediated by inhibition of virus replication by production of IFNγ and by MHC class II restricted cytotoxicity [40]. Type I IFNs can promote expansion of CD4^+ ^T cells either indirectly through effects on antigen-presenting cells [42] or through direct action [43,44]. The current findings suggest that co-delivery of certain type I IFN subtypes in our vaccine may facilitate the induction of direct anti-viral CD4^+ ^T cell activity. This is supported by the fact that we see a strong correlation of high control of FV-induced disease with the augmented CD4^+ ^T cell response, whereas the levels of binding and neutralizing antibodies are not equally increased by co-administration of all analyzed IFNs, indicating a direct function of virus-specific CD4^+ ^T cells.

In the FV model, the only known CD8^+ ^T cell epitope is located in the leader region of the Gag protein [21], but after vaccination with adenoviral vectors encoding F-MuLV Gag, no immune response against this epitope was detected because the epitope peptide was obviously not processed. However, the vaccination with adenoviral vectors very likely induced CD8^+ ^T cell responses of up to now unknown specificity as activated CD8^+ ^T cells with effector phenotype were detected after immunization (data not shown). This is in line with previous reports that after vaccination with vaccinia viruses encoding different F-MuLV Gag constructs also regions other than the leader can induce protective CD8^+ ^T cell immune responses [45]. However, our CD8^+ ^T cell depletion experiment did not result in reduced protection, suggesting that CD8^+ ^T cell responses against other epitopes did not play a critical role for the improved protection after co-administration of type I IFNs. Our data from vaccination of mice against HIV proteins also showed only minor effects of type I IFNs on CD8^+ ^T cell responses, suggesting that these cytokines may not be very efficient to enhance adenovirus-based vaccine induced CD8^+ ^T cell responses.

Some of the IFN subtypes used in this study have been evaluated before to enhance immune responses to protein, plasmid or viral vector based vaccines. The data imply that the adjuvant potential of IFN subtypes may vary depending on the type of vaccine. For DNA and protein-based vaccines against model antigens or virus infections, most efforts to improve vaccine efficacy by co-administration of type I IFNs resulted in improved antibody and CD8^+ ^T cell responses that mediated enhanced protection [8,26-30]. The effect of type I IFN co-expression in virus-vector based vaccines, however, seems to depend on the vector-type. Immune responses, but not protection, induced by a rabies virus based vaccine against HIV could be improved by co-expression of IFNβ [32]. The protective effect of a vaccinia virus-vectored vaccine against influenza, on the other hand, was not improved by IFNβ or IFNα4 co-administration [31]. Interestingly, none of the above cited publications reports an enhanced induction of CD4^+ ^T cells, while we found strongly augmented CD4^+ ^T cell responses that correlated well with the observed improved protection against systemic FV challenge infection conferred by codaministration of specific type I IFN subtypes with the vaccine.

It seems plausible that virus-based vectors are more immunogenic by themselves and thus effects of cytokine coexpression are harder to achieve than with DNA or protein vaccines. In our current vaccination study, the finding that only IFNα2, IFNα4, IFNα6 and IFNα9, but not IFNα1, IFNα5 and IFNβ, had an adjuvant effect on the adenovirus-based vaccine may also be related to the immunogenicity of the vector itself. In fact, the ineffective IFN subtypes were those that were expressed at the highest levels in Ad-infected DCs (see Additional file 2, Figure S2), which is in accordance with an earlier report showing that the adenovirus-induced type I IFN response was predominantly comprised of IFNα1, IFNα5 and IFNβ [46]. While additional expression of these IFN subtypes from the vaccine vectors did not enhance immunogenicity, broadening the IFN profile by expressing other IFN subtypes seems to be more effective. It has been established that, while all type I IFNs signal through the same receptor, the IFN subtypes bind this receptor with different affinities [47,48]. These differences in receptor binding can result in different downstream signaling events and distinct induction of interferon stimulated genes. Thus, the tested IFN subtypes may have induced different activation pattern in vaccine-primed immune cells, which might be an underlying mechanism for their distinct adjuvant effects.

The homology between murine and human type I interferons is rather low with about 70-75% at the nucleotide level [49], so a direct translation of our findings into HIV vaccine development for humans is difficult. However, also human type I interferon subtypes exhibit distinct immunological functions [47], so that our main findings that different IFN subtypes have distinct potencies as vaccine adjuvants should hold true for human IFN subtypes as well. Thus, human IFN subtypes should be tested when developing HIV prototype vaccines.

We demonstrated that the protective effect of low-dose immunization against retroviruses with adenoviral vectors could be highly improved by co-administration of specific vectored type I IFN subtypes, which induced strong CD4^+ ^T cell responses and enhanced binding antibody titers. This shows that careful manipulation of the cytokine milieu can result in impressive advancement in vaccine efficacy and suggests that IFN subtypes may be useful tools to improve immune responses to adenovirus-based vaccines.

## Conclusions

This study examines the adjuvant effect of distinct type I IFN subtypes on an adenoviral vector-based anti-retroviral vaccine. In the Friend virus model, the co-delivery of IFNα2, IFNα4, IFNα6 and IFNα9 together with viral antigens by an adenovirus-based vaccine resulted in strong improvement of vaccine efficacy that was apparent by high control over FV-induced disease and correlated with improved CD4^+ ^T cell responses and higher binding antibody titers. Similar findings were made when mice were immunized with adenovirus-based vectors encoding HIV proteins. No influence on vaccine efficacy was observed for co-administration of vectored IFNα1, IFNα5 and IFNβ. Our results show that co-expression of specific type I IFN subtypes should be considered for adenovirus-based anti-retroviral vaccination.

## Methods

### Cells and cell culture

The human embryonic kidney cell line 293 (Microbix Biosystems, Toronto, ON, Canada), the human lung carcinoma cell line A549 (ATCC # CCL-185) and the type I IFN indicator cell line MxRAGE7 ([20], Werner Müller, German Research Centre for Biotechnology, Braunschweig, Germany) were propagated in Dulbecco's modified Eagle medium (DMEM) with high glucose. A murine fibroblast cell line from *Mus dunni *[50] was maintained in RPMI medium (Invitrogen/Gibco, Karlsruhe, Germany). Cell culture media were supplemented with 10% heat-inactivated fetal bovine serum (Invitrogen/Gibco) and 50 μg/ml gentamicin. Cell lines were maintained in a humidified 5% CO_2 _atmosphere at 37°C (293, *M. dunni*) or 32°C (MxRAGE7).

### Adenoviral vectors

The adenoviral vectors Ad5.env, Ad5F35.env, Ad5.gag, and Ad5F35.gag [18] encode full-length F-MuLV Env or Gag proteins amplified by PCR from F-MuLV clone FB29 [51]; vectors were obtained using the AdEasy system and vectors pAdTrackCMV, pAdEasy-1, and pAdEasy-1/F35.

The adenoviral vector Ad5.Henv contains a codon-optimized full-length *env *based on HIV clade C isolate CN54 and was obtained using the AdEasy system after cloning of synthesized DNA into pShuttle-CMV plasmid. Ad5.Hgpsyn contains a codon-optimized HIV *gag-pol *based on the HIV clade B isolate BH10 that was described before [52] and was constructed using the AdEasy system and vectors pShuttle-CMV and pAdEasy-1.

The adenoviral vectors Ad5.Luc and Ad5F35.Luc encoding firefly luciferase have been described before [53,54].

For the construction of type I IFN encoding adenoviral vectors, cDNAs for IFNα1, IFNα2, IFNα4, IFNα5, IFNα6, IFNα9 and IFNβ were subcloned from the plasmids pkCMVint.IFNα1, pkCMVint.IFNα2, pkCMVint.IFNα4, pkCMVint.IFNα5, pkCMVint.IFNα6, pkCMVint.IFNα9 [4] and pkCMVint.IFNβ [5] into pShuttle, recombinant Ad5 and Ad5F35 based vectors were obtained by homologous recombination of pShuttle constructs with pAdEasy-1 and pAdEasy-1/F35, respectively, and transfection into 293 cells as described before [18].

All adenoviral vectors were purified with the Vivapure AdenoPACK 100 kit (Vivascience, Hannover, Germany). The adenovirus particle concentrations were determined by spectrophotometry as described previously [55] and expressed as viral particles (VP)/ml. The particle-to-PFU ratio of all vector preparations was ~30:1.

Equal expression levels of IFNs by the recombinant adenovirus constructs were verified by a cell based bioassay using MxRAGE7 cells as described before [20]. Briefly, non-complementing A549 cells were transduced with type I IFN encoding adenoviral vectors at an MOI of 100, culture supernatants were collected three days p.i. and added to subconfluent MxRAGE7 cells that were incubated for 2 days at 37°C. Type I IFN induced GFP expression was analyzed by flow-cytometry.

### Mice

Female CB6F1 hybrid mice (BALB/c × C57BL/6 F1; H-2^b/d ^Fv1^b/b ^Fv2^r/s ^Rfv3^r/s^) and female BALB/c mice were purchased from Charles River Laboratories (Sulzfeld, Germany). All mice were used when they were between 8 and 9 weeks of age and were treated in accordance with the regulations and guidelines of the institutional animal care and use committee of the Ruhr University Bochum, Germany.

### Immunization

For immunization against FV, CB6F1 mice were immunized with Ad5 and Ad5F35-based vectors using a heterologous prime-boost immunization protocol with a 21-day interval. 1 × 10^9 ^VP each of F-MuLV Env- and Gag-encoding vectors were mixed with either 1 × 10^9 ^VP of a type I IFN-encoding vector or a luciferase-encoding vector as a control and injected into both hind footpads in 100 μl PBS. Ad5 vectors were used for the prime immunizations, and fiber-chimeric Ad5F35 vectors were used for boost immunizations.

In a control experiment, CB6F1 mice were immunized as described above with 1 × 10^9 ^VP of type I IFN-encoding vectors alone, using Ad5 vectors for prime and Ad5F35 vectors as boost immunizations.

To deplete CD4^+ ^or CD8^+ ^cells around the time of vaccination, mice were injected intraperitoneally with the antibodies 191.1 or 169.4 [56], respectively, on days -3, -1, +1, +3, and +5 around the day when the vaccine was applied. The depletion was performed when mice were prime- and boost-immunized.

For immunization against HIV, BALB/c mice were vaccinated with a single or a prime-boost immunization with Ad5-based vectors encoding HIV Env and Gag-Pol mixed with type I IFN- or luciferase-encoding vectors as described for vaccination against FV. When mice were boost immunized, Ad5-based HIV Env- and Gag-Pol-encoding vectors and Ad5F35-based type I IFN- or luciferase-encoding vectors were used.

### FV and challenge infection

Uncloned, lactate dehydrogenase-elevating virus (LDV)-free FV stock was obtained from BALB/c mouse spleen cell homogenate (10%, wt/vol) 14 days p.i. with a B-cell-tropic, polycythemia-inducing FV complex [57]. CB6F1 mice were challenged by the intravenous injection of 250 spleen focus-forming units. The course of disease was monitored twice a week by palpation of the spleen of each animal under general anesthesia. The spleen size was rated on a scale ranging from 1 (normal spleen size) to 4 (severe splenomegaly), as described previously [58].

### Viremia assay

Ten days post challenge (p.c.), plasma samples from CB6F1 mice were obtained, and viremia was determined in a focal infectivity assay [59]. Serial dilutions of plasma were incubated with *M. dunni *cells for 3 days under standard tissue culture conditions. When cells reached ~100% confluence, they were fixed with ethanol, labeled with F-MuLV Env-specific MAb 720 [60], and then with a horseradish peroxidase (HRP)-conjugated rabbit antimouse Ig antibody (Dako, Hamburg, Germany). The assay was developed using aminoethylcarbazole (Sigma-Aldrich, Deisenhofen, Germany) as substrate to detect foci. Foci were counted, and focus-forming units (FFU)/ml plasma were calculated.

### Infectious center assay

21 days p.c. FV-infected animals were sacrificed by cervical dislocation, the spleens were removed and weighed, and single-cell suspensions were prepared. Serial dilutions of isolated spleen cells were seeded onto *M. dunni *cells and incubated under standard tissue culture conditions for 3 days, fixed with ethanol, and stained as described for the viremia assay. Resulting foci were counted, and infectious centers (IC)/spleen were calculated.

### Binding antibody ELISA

For the analysis of F-MuLV-binding antibodies, MaxiSorp ELISA plates (Nunc, Roskilde, Denmark) were coated with whole F-MuLV antigen (5 μg/ml), blocked with fetal calf serum, and incubated with serum dilutions. Binding antibodies were detected using a polyclonal rabbit-anti-mouse HRP-coupled anti-IgG antibody and the substrate tetramethylbenzidine (TMB+; both Dako Deutschland GmbH, Hamburg, Germany). Sera were considered positive if the optical density at 450 nm was 3-fold higher than that obtained with sera from naïve mice.

### Complement-dependent F-MuLV-neutralizing antibody assay

To detect F-MuLV-neutralizing antibodies, serial dilutions of plasma in PBS were mixed with purified F-MuLV and guinea pig complement (Institut Virion/Serion GmbH, Wuerzburg, Germany), incubated at 37°C for 60 min, and then added to *M. dunni *cells that had been plated at a density of 7.5 × 10^3 ^cells per well in 24-well plates the day before. Seventy-two hours later cells were stained as described for the viremia assay. Dilutions that resulted in a reduction of foci by 50% or more were considered neutralizing.

### Tetramer staining of F-MuLV-specific T cells

Spleens of CB6F1 mice were removed 3 days post-challenge (p.c.), and single-cell suspensions were prepared. For analysis of CD4^+ ^T cells, spleen cells were stained with a phycoerythrin (PE)-coupled major histocompatibility complex (MHC) class II tetramer (containing the I-Ab-restricted F-MuLV Env epitope EPLTSLTPRCNTAWNRLKL [22]; kindly provided by the MHC Tetramer Core Facility of the National Institutes of Health, National Institute of Allergy and Infectious Disease, Atlanta, GA), peridinin chlorophyll protein (PerCP)-anti-CD4, and fluorescein isothiocyanate (FITC)-anti-CD11b (Becton Dickinson, Heidelberg, Germany). For detection of virus-specific CD8^+ ^T cells, spleen cells were stained with PE-coupled MHC I tetramer (containing the H-2Db restricted F-MuLV Gag-leader epitope AbuAbulLAbuLTVFL in which cysteine residues of the original amino acid sequence were replaced by amino-butyric acid to prevent disulfide bonding [21]), allophycocyanin (APC)-anti-CD8 and FITC-anti-CD43. Data were acquired on a flow cytometer (FACSCalibur; Becton Dickinson, Mountain View, CA) and analyzed using CellQuest Pro (version 4.0.1; Becton Dickinson) and FlowJo (version 7.6; Tree Star, Ashland, OR) software.

### Flow-cytometric analysis of T cell induction

To analyze the induction of T cells by the vaccine, mice were immunized once by footpad injection with Ad5.env and Ad5.gag vectors (1 × 10^9 ^VP each) and co-immunized with 1 × 10^9 ^VP Ad5.IFNα4 or Ad5.GFP as a control. Ten days later, the popliteal lymph nodes were isolated and lymph node cells were analyzed by flow cytometry. For analysis of the activation of CD4^+ ^cells, we used the antibodies PE-anti-CD4 (Becton Dickinson), peridinin chlorophyll protein complex (PerCP)-anti-CD43 (BioLegend, Fell, Germany), phycoerythrin-cyanin-7 (Pe-Cy7)-anti-CD62L (eBioscience, Frankfurt, Germany), APC-anti-CD44 (Becton Dickinson) and eFluor450-anti-CCR7 (eBioscience). Data were acquired on an LSR II flow cytometer (Becton Dickinson) and analyzed using FlowJo software (Tree Star).

### Intracellular cytokine staining

HIV-specific T cells were characterized by intracellular cytokine staining. Two weeks after immunization with vectors encoding HIV Env and Gag-Pol, spleens were removed and spleen cells were stimulated for 6 h *in vitro *with HIV Env- or Gag-derived peptides (IHIGPGRAFYT, gp120_309-320 _[61], AMQMLKETI p24_65-73 _[24]) or Gag-derived peptides (SPEVIPMFSALSEGA, p24_165-179_, PVGEIYKRWIILGLN, p24_257-271 _[25]; Metabion, Martinsried, Germany) representing described CD8^+ ^and CD4^+ ^T cell epitopes, respectively. Cells were stained with PE-anti-interferon gamma (IFN-γ), APC-anti-interleukin-2 (IL-2), FITC-anti-tumor necrosis factor alpha (TNF-α) and either PerCP-anti-CD8 or PerCP-anti-CD4 (all from Becton Dickinson, Heidelberg, Germany) and analyzed by flow cytometry.

### Statistical analyses

Statistical analyses were performed using the software SigmaStat 3.1 (Systat Software GmbH, Erkrath, Germany), testing with the Kruskal-Wallis one-way analysis of variance on ranks and Student-Newman-Keuls multiple comparison procedure.

## Competing interests

The authors declare that they have no competing interests.

## Authors' contributions

WB constructed viral vectors encoding FV antigens, participated in the study design and drafted the manuscript. RL constructed viral vectors encoding IFNs and carried out the animal experiments. TO constructed viral vectors encoding IFNs. LJ performed part of the animal experiments. MT performed flow cytometric analyses. GN produced and characterized the viral vectors encoding HIV antigens. SS participated in FV experiments. PG constructed viral vectors encoding HIV antigens. HW provided essential material for the study and revised the manuscript. CB provided essential material to conduct the study and revised the manuscript. KÜ participated in the study design. UD conceived the study, participated in its design, analyzed data, and wrote the manuscript. OW designed the study, generated some of the adenoviral IFN encoding vectors, analyzed data, and revised the manuscript. All authors read and approved the final manuscript.

## Supplementary Material

Additional file 1 **Figure S1: Immunization schemes**. This additional file provides schematic layouts of the experiments, indicating treatment and analysis schedules. Click here for file

Additional file 2 **Figure S2: Expression levels of type I interferons in Ad-infected DCs**. The intrinsic expression levels of the tested type I interferons in DCs infected with Ad5.env were analyzed and compared to uninfected DCs. Click here for file
