# Supplementary material for: Improved vaccine protection against retrovirus infection after co-administration of adenoviral vectors encoding viral antigens and type I interferon subtypes
Source: Retrovirology. 2011 Sep 26;8:75. doi: 10.1186/1742-4690-8-75 (PMC3193818; doi:10.1186/1742-4690-8-75)
Supplement: Additional file 1 — Figure S1: Immunization schemes. This additional file provides schematic layouts of the experiments, indicating treatment and analysis schedules. [file 1742-4690-8-75-S1.PDF]

Supplementary Fig. 1 - Immunization schemes

A - FV challenge experiment (Fig. 1, Fig. 2, Fig. 5)

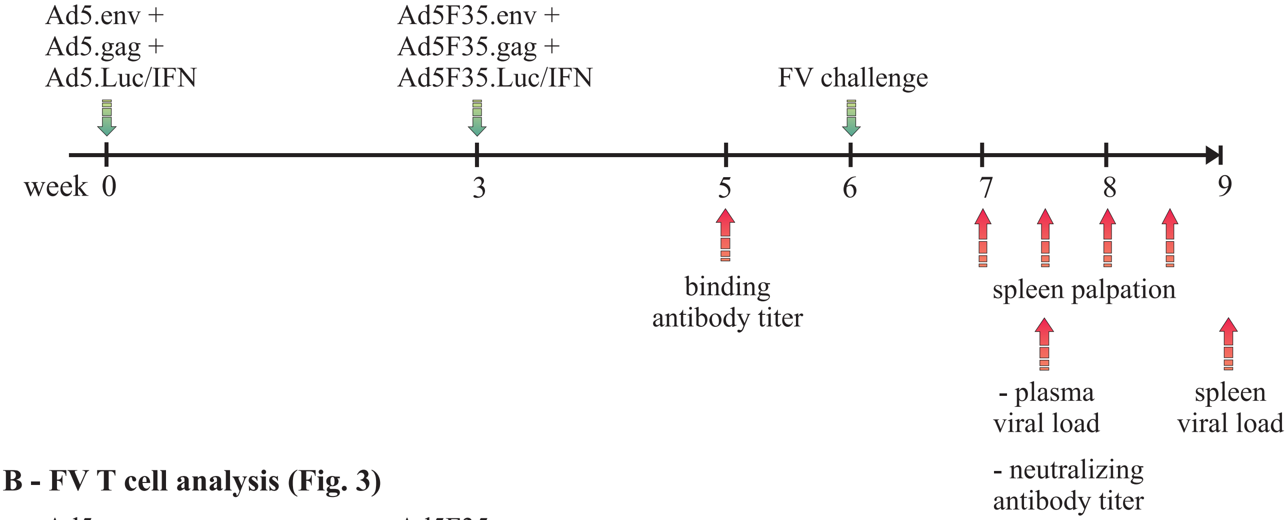

B - FV T cell analysis (Fig. 3)

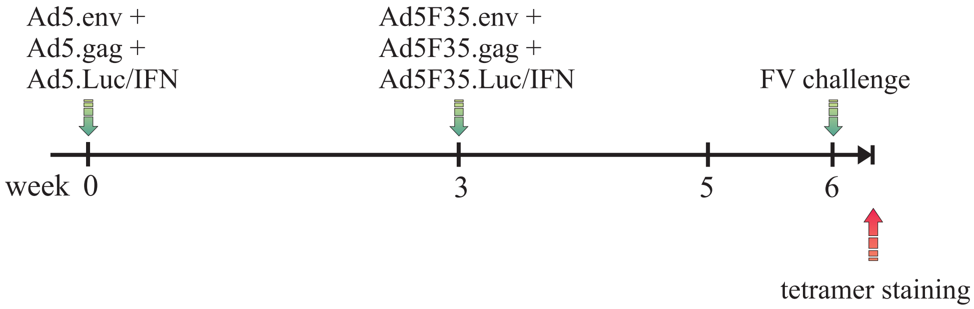

C - FV depletion experiment (Fig. 4)

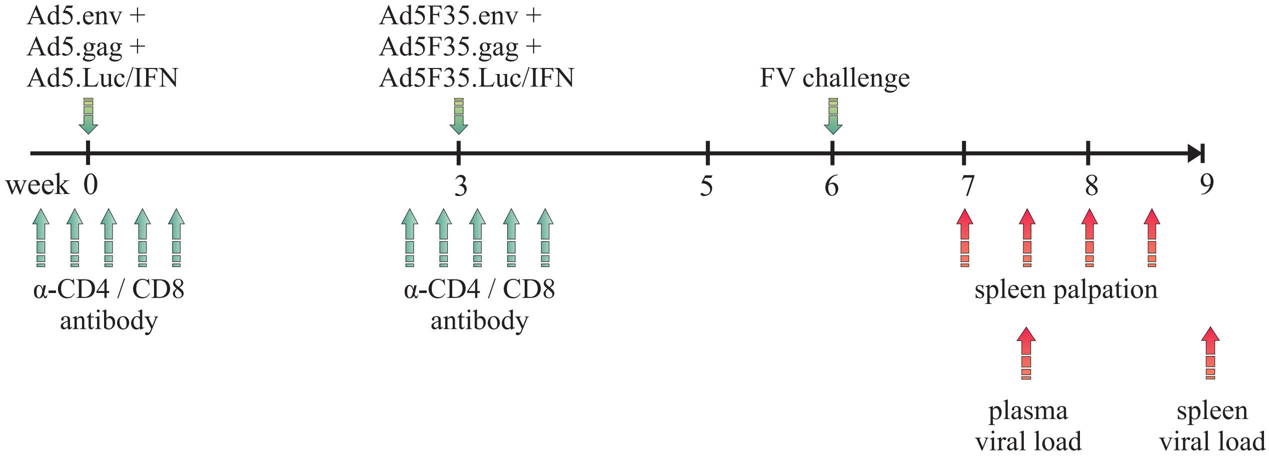

D - HIV vaccination T cell analysis (Fig. 6)

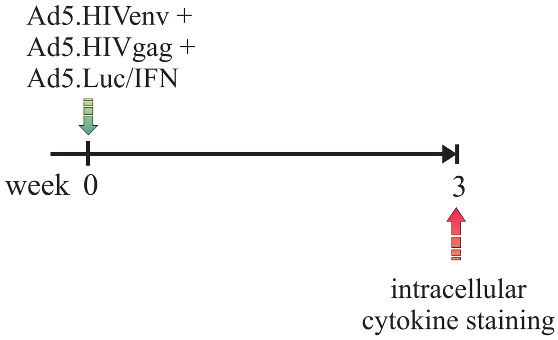

Immunization schemes
Schematic representation of the experimental layouts. Indicated are the treatments and assays performed in the experiments. The corresponding data is shown in the indicated figures of the manuscript.
