# Supplementary material for: Improved vaccine protection against retrovirus infection after co-administration of adenoviral vectors encoding viral antigens and type I interferon subtypes
Source: Retrovirology. 2011 Sep 26;8:75. doi: 10.1186/1742-4690-8-75 (PMC3193818; doi:10.1186/1742-4690-8-75)
Supplement: Additional file 2 — Figure S2: Expression levels of type I interferons in Ad-infected DCs. The intrinsic expression levels of the tested type I interferons in DCs infected with Ad5.env were analyzed and compared to uninfected DCs. [file 1742-4690-8-75-S2.PDF]

Supplementary Fig. 2 - Expression levels of type I interferons in Ad-infected DCs

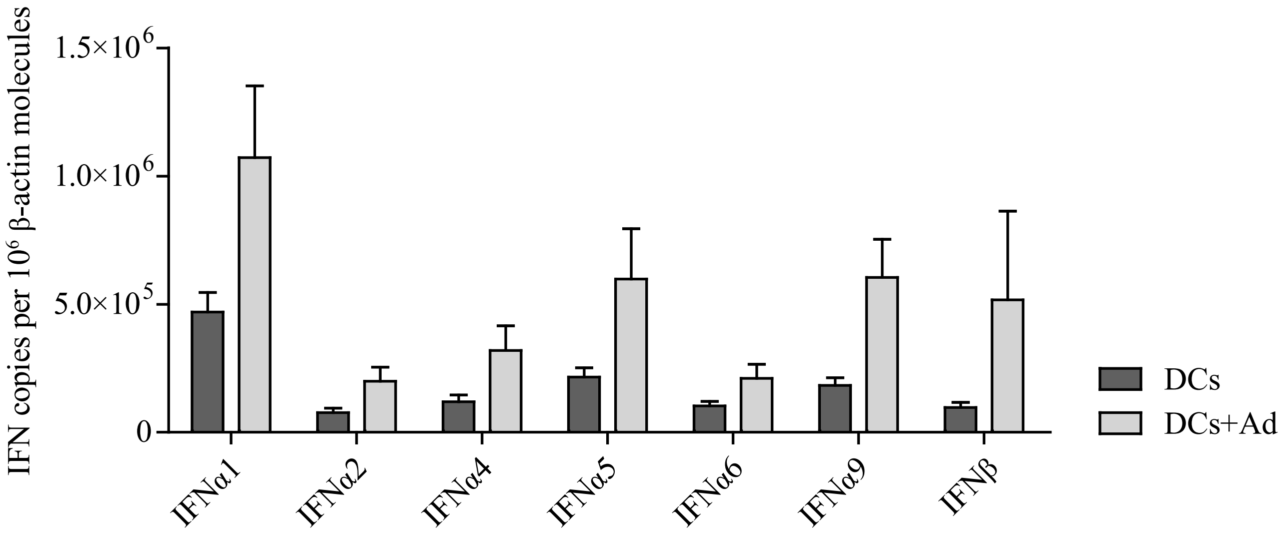

Expression levels of type I interferons in Ad-infected DCs

Dendritic cells (DCs) were isolated from lymph nodes of a naïve mouse by MACS sorting using CD11c beads (Miltenyi Biotec, Bergisch-Gladbach, Germany) and infected with Ad5.env at an MOI of 1000. RNA was isolated from Ad5.env-infected and uninfected control DCs after 6h cultivation using Trizol reagent (Invitrogen, Karlsruhe, Germany). Primers specific for the murine interferons IFNα1, IFNα2, IFNα4, IFNα5, IFNα6, IFNα9 and IFNβ (Quanti-Tect Primers, Qiagen, Hilden, Germany) and murine β-actin (Miller et al., Differentiation 2003) were used in SYBR Green based RT-PCR reactions (Power SYBR Green 1-Step-Kit, Applied Biosystems, Darmstadt, Germany) on a StepOnePlus Real-Time PCR cycler (Applied Biosystems) as described before (Gerlach et al., Eur. J. Immunol. 2009) and copy numbers of type I interferons were calculated relative to β-actin molecules.
